# AI for mental health: clinician expectations and priorities in computational psychiatry

**DOI:** 10.1186/s12888-025-06957-3

**Published:** 2025-06-06

**Authors:** Leo Fischer, Paula Antonia Mann, Minh-Hieu H. Nguyen, Stefan Becker, Shiva Khodadadi, Antonia Schulz, Sharmili Edwin Thanarajah, Jonathan Repple, Tim Hahn, Andreas Reif, Amir Salamikhanshan, Sarah Kittel-Schneider, Winfried Rief, Christoph Mulert, Stefan G. Hofmann, Udo Dannlowski, Tilo Kircher, Felix P. Bernhard, Hamidreza Jamalabadi

**Affiliations:** 1 Department of Psychiatry and Psychotherapy, University of Marburg, Marburg, Germany; 2 Department of Psychiatry, Psychosomatic Medicine and Psychotherapy, University Hospital Frankfurt, Frankfurt, Germany; 3 Institute for Translational Psychiatry, University of Münster, Münster, Germany; 4 Fraunhofer Institute for Translational Medicine and Pharmacology ITMP, Frankfurt am Main, Frankfurt, Germany; 5 Department of Psychiatry and Neurobehavioural Science, University College Cork, Cork, Ireland; 6 APC Microbiome, University College Cork, Cork, Ireland; 7 Department of Psychiatry, Psychotherapy and Psychosomatic Medicine, University Hospital Würzburg, Würzburg, Germany; 8 Department of Psychology, University of Marburg, Marburg, Germany; 9 Centre of Psychiatry, Justus Liebig University Giessen, Giessen, Germany; 10 Center for Mind, Brain, and Behavior (CMBB), University of Marburg, Marburg, Germany; 11 Cooperative Brain Imaging Center - CoBIC, Goethe University Frankfurt, Frankfurt, Germany

**Keywords:** Computational psychiatry, Ecological momentary assessment (EMA), AI, Clinician expectations

## Abstract

Mental disorders represent a major global health challenge, with an estimated lifetime prevalence approaching 30%. Despite the availability of effective treatments, access to mental health care remains inadequate. Computational psychiatry, leveraging advancements in artificial intelligence (AI) and machine learning (ML), has shown potential for transforming mental health care by improving diagnosis, prognosis, and the personalization of treatment. However, integrating these technologies into routine clinical practice remains limited due to technical and infrastructure challenges. While ongoing computational developments will enhance AI’s precision, many studies focus on its broad potential without providing specific, clinician-informed guidance for immediate application. To address this gap and the urgent need for clinically actionable AI tools, we surveyed 53 psychiatrists and clinical psychologists to identify their priorities for AI in mental health care. Our findings reveal a strong preference for tools enabling continuous monitoring and predictive modeling, particularly in outpatient settings. Clinicians prioritize accurate predictions of symptom trajectories and proactive patient monitoring over interpretability and explicit treatment recommendations. Self-reports, third-party observations, and sleep quality and duration emerged as key data inputs for effective models. Together, this study provides a clinician-driven roadmap for AI integration, emphasizing predictive models based on ecological momentary assessment (EMA) data to forecast disorder trajectories and support real-world practice.

**Supplementary Information:**

The online version contains supplementary material available at 10.1186/s12888-025-06957-3.

## Introduction

Mental disorders pose a significant global public health challenge, with lifetime prevalence rates approaching 30%, making them a leading cause of disability, increased mortality, and lost workdays worldwide [1, 2]. While effective treatments exist, such as combinations of psychotherapy and medication, access to these treatments remains limited. For instance, approximately 77% of counties in the United States lack adequate psychiatric care [3], and patients in Germany often face waiting times exceeding three months for an initial consultation with a psychotherapist [4]. In low-income countries, mental health resources are up to 100 times scarcer than in wealthier nations [5]. Addressing these disparities is a crucial public health priority [1, 6].

Unlike other clinical conditions with clear symptoms like fever or pain, prevalent mental disorders manifest through symptoms such as sadness, guilt, and changes in appetite, which can be mistakenly perceived as part of normal life [7]. Consequently, many individuals delay seeking help until symptoms have fully developed, compounding the challenge for clinicians, who are often limited in their capacity to provide timely care [8]. Psychotherapy, for example, typically requires a minimum of six months, and some forms may last years, severely limiting clinician availability [9]. Even pharmacological treatments demand close supervision to ensure effectiveness, with over 50% of patients needing a medication change within the first eight weeks [10]. Mental health disorders are often characterized by high relapse rates, with over 50% of cases recurring within one year. This recurrence adds a substantial clinical burden that cannot be addressed solely by increasing clinical staff [11].

In this context, computational psychiatry-applying artificial intelligence (AI) and machine learning (ML) to mental health-offers a promising avenue for addressing the complexities of psychiatric disorders. By modeling the interplay between biological, psychological, and social factors, AI-driven approaches can potentially facilitate earlier diagnosis, improve prognostic accuracy, personalize treatment, and even accelerate new therapeutic developments [12–19]. Moreover, advancements in smartphone and web-based technologies have enabled efficient data collection, potentially easing clinician and patient burdens [20–22]. Despite these advancements, the integration of computational psychiatry in routine practice remains limited, with challenges such as model complexity and a perceived gap between AI research and practical applicability. Major studies even question the feasibility of accurate treatment predictions, citing significant limitations in current methodologies [23].

Given the pressing need for actionable solutions, computational psychiatry research should prioritize applications with immediate translational value while simultaneously advancing AI’s predictive accuracy. Providing support to clinicians should happen thus in parallel with ongoing methodological improvements. While prior studies have outlined AI’s potential in mental health (e.g., [13, 24, 25]) or surveyed general attitudes (e.g., [26]), few offer empirical, clinician-driven insights into specific, immediately actionable functionalities. Our study seeks to clarify clinicians’ initial expectations regarding the role of AI in mental health practice by capturing their perspectives. Through a survey of 53 psychiatrists and clinical psychologists, we identified the AI functionalities they find most valuable and the data types they consider essential for robust model training. By aligning computational psychiatry research with clinicians’ immediate needs, our findings provide a targeted foundation for developing AI tools that effectively support mental health professionals, bridging the gap between technological innovation and clinical application [12].

## Methods

### Data acquisition

To design a structured questionnaire for the current study, we began with semi-structured interviews (see Supplementary A) conducted with eight clinicians. These interviews aimed to capture clinicians’ insights, including their expectations, concerns, and overall perspectives on the application of AI in mental health practice. The insights from these discussions informed the design and focus of our final questionnaire, allowing us to prioritize key topics relevant to clinical needs. The interview questions were designed to be open-ended and neutral (e.g., “What are the potential applications of AI in a psychiatric context?”) to minimize bias and avoid guiding participants toward specific responses. Conducted by LF and PM, the interviews followed a flexible protocol, with probing used only for clarification, ensuring responses reflected clinicians’ unprompted views. Interview participants were primarily recruited from the Department of Psychiatry and Psychotherapy at the University Hospital Marburg and included a diverse group: a neurologist, a psychiatrist, four psychiatric residents, a psychologist, and a psychologist in training as a psychotherapist. These preliminary interviews were essential to ensure that the questionnaire reflected perspectives from outside the author team, further allowing us to validate that our questions were comprehensive and applicable across different clinical contexts. We conducted a qualitative analysis of the interview responses to extract meaningful themes. Themes were identified through qualitative content analysis, independently coded by LF and PM, and reviewed jointly to reach consensus. This process involved iteratively categorizing open-ended responses into themes (e.g., prediction utility, trust in AI) through discussion, ensuring all relevant clinician perspectives were captured.

Based on these qualitative insights, we developed a 24-item questionnaire (see Supplementary B) to further explore the identified themes. The identified themes-such as the importance of predictive accuracy and preferred data inputs-were directly mapped onto the questionnaire items. For example, the emphasis on prediction utility informed items assessing the relevance of specific prediction details, while trust concerns shaped accuracy threshold questions. The draft questionnaire was subsequently piloted with a subset of interviewees to ensure items accurately captured their expressed perspectives, refining wording as needed. This questionnaire was then distributed to a larger group of clinicians, including German-speaking clinical psychologists and psychiatrists based in Germany and Ireland. Given the exploratory nature of our research, we targeted a sample size of 60 participants, ensuring a balanced representation across various professional roles and levels of clinical experience. This target aligns with sample sizes in similar exploratory studies of clinician perspectives on AI in mental health (e.g., Weermeijer et al., 2024 [27]), prioritizing depth of insight from practicing clinicians over large-scale statistical power. We employed a purposive snowball sampling strategy, initiating recruitment through our professional networks and expanding via referrals, resulting in 45 survey respondents and a total sample of 53 clinicians (including interviewees). This approach ensured participants were actively engaged in clinical practice and represented diverse settings, including major mental health consortia in Europe and a smaller subset from the USA. By"various professional roles,"we refer to distinct occupational categories within mental health care: fully qualified psychologists, psychologists in training, licensed psychotherapists, psychiatric residents, and fully qualified psychiatrists, as well as a neurologist in the interview phase, capturing both medical and psychological expertise across training stages. Data collection was facilitated using the SosciSurvey platform. The study protocol was reviewed and approved by the Ethics Committee of Philipps University Marburg, with no ethical concerns raised.

The final sample had a gender distribution of 60% identifying as female, 37.8% as male, and 2.2% as diverse. Participant ages ranged from 23 to 81 years, with a mean age of 42 years (SD = 12.9). This sample included a wide array of mental health professionals: four psychologists, four psychologists in training, 15 psychotherapists, six psychiatric residents, and 16 psychiatrists. Clinical experience among respondents varied significantly, ranging from less than one year to over 21 years.

### Statistical analysis

Descriptive statistics were initially calculated for key demographic groups (e.g., gender, psychology vs. medical background, clinical experience of less than vs. more than ten years, and university vs. other professional settings) and for each Likert-scale item on the questionnaire. To identify potential differences in responses across these groups, Mann-Whitney U tests were conducted with a significance threshold of $p < 0.05$, chosen due to the ordinal nature of our Likert-scale data, which precludes parametric assumptions of normality [28]. Missing data from two incomplete survey responses (N=2 out of 45) were handled by excluding these cases from relevant statistical tests to preserve data integrity. Additionally, responses with missing data on individual questionnaire items ($N = 2$) were excluded from the associated statistical tests.

## Results

We first sought to understand the extent of clinicians’ training and familiarity with computational sciences (AI, ML) in psychiatric contexts. The results revealed that most clinicians surveyed had limited exposure to AI: 44.4% reported no formal knowledge, while 33.3% indicated only minimal familiarity. None of the eight interviewed clinicians had hands-on experience using AI tools, although three reported encountering AI research in clinical settings.

**Fig. 1:**
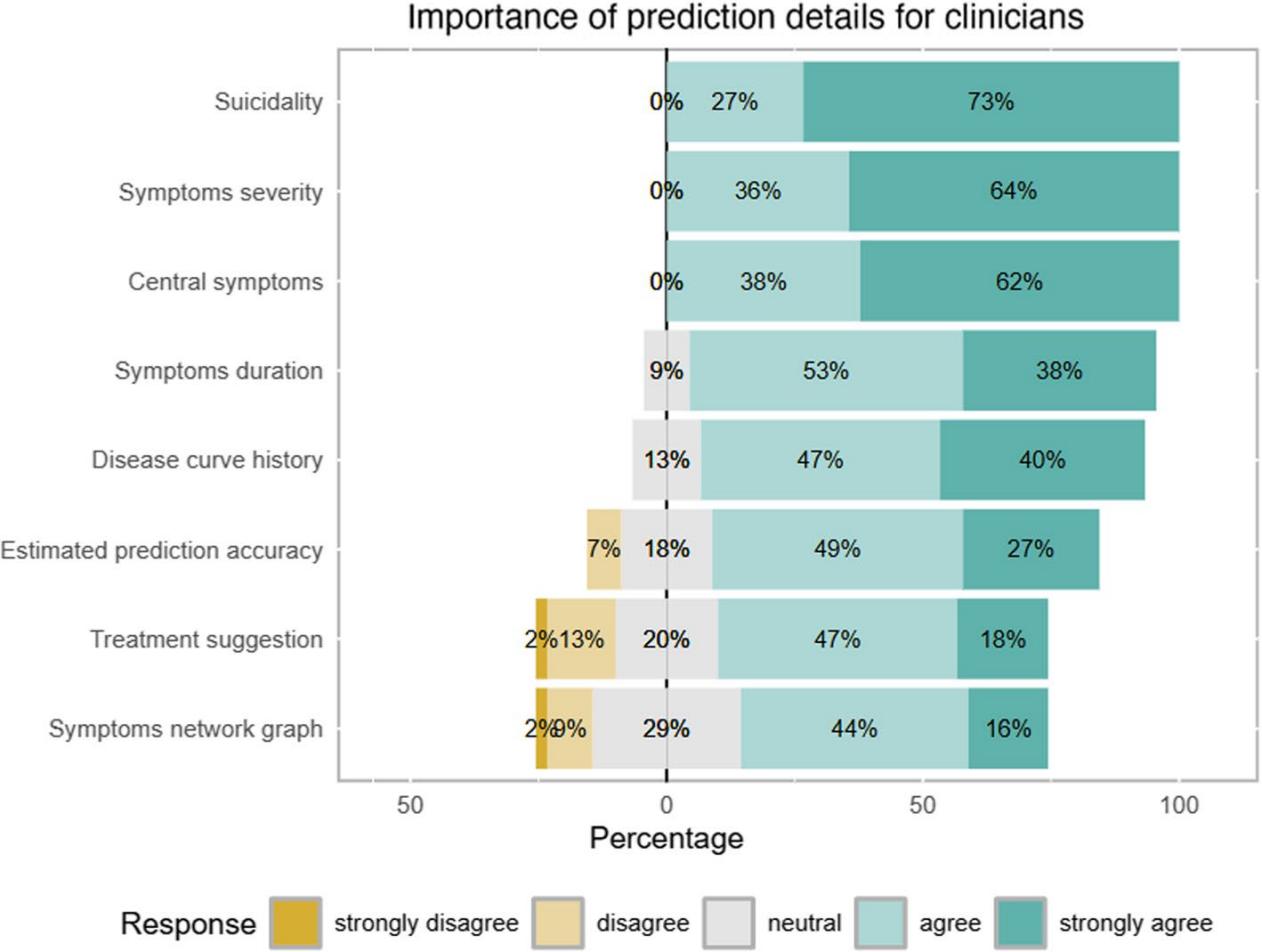
Clinician perspectives on the importance of various prediction details in AI-assisted mental health tools. The chart illustrates the percentage of clinicians who agreed or disagreed with the relevance of different prediction elements. High priority is given to details about *suicidality*, *symptom severity*, *central symptoms*, *symptom duration*, and *disease curve history*. Prediction accuracy is also valued, while *treatment suggestions* and *symptom network graphs* are regarded as less critical. The responses highlight a clear preference for monitoring and prediction over interpretability and treatment guidance, underscoring the importance of actionable, patient-specific insights where mainly the indicating stronger support for AI enhancing therapy decisions compared to specific recommendations

**AI for symptom prediction and information preferences for prediction outcomes**. Clinicians identified that the primary role of AI should center around reliable predictions, particularly concerning patient suicidality (73% “*very important*”), symptom severity (64% “*very important*”), and the central symptoms (62% “*very important*”) stabilizing depression (Fig. 1). These elements were rated as essential for effective patient monitoring and support. In contrast, findings from the semi-structured interviews revealed relatively low interest in therapy recommendations (18% rated them as “very important”), network visualizations (16% rated them as “very important”), and exact prediction accuracy (27% rated them as “very important”). These results highlight clinicians’ preference for a quick, intuitive overview of patient risk over detailed, structured information. Additionally, clinicians highlighted sleep (sleep quality: 47% “*very well suited*”, 51% “*rather suitable*”; sleep duration: 24%/49%) problems as the most relevant supplementary parameter for improving prediction accuracy, showing strong confidence in its utility for patient assessment. Motor activity (8,9% “*very well suited*”, 60% “*rather suitable*”) and dietary habits (2%/42%) were also seen as valuable, though to a lesser extent. Notably, there was a significant divide on the value of heart rate as a parameter: university-affiliated clinicians viewed it as beneficial, while non-university clinicians were more skeptical (U = 218.5; Z = 3.51; $p < 0.0004$).

Differences also emerged between professional backgrounds: psychiatrists were generally more inclined to disclose prediction outcomes to patients than clinical psychologists, with physicians showing a higher willingness to share information (U = 324.5; Z = 2.14; $p <0.032$). Additionally, university-affiliated clinicians placed greater importance (*mean rank* = 22.8636) in saving time through use of AI support than their non-university counterparts (*mean rank* = 14.9348) (U = 185.5; Z = 2.2397; $p < 0.025$).

**AI utility in clinical settings**. Clinicians indicated that the highest utility for AI in psychiatric settings would likely be in outpatient environments, where crisis prevention and therapeutic monitoring are primary goals (73.4% “very important” for outpatient settings). This preference was particularly among psychiatrists and clinical psychologists, who considered AI-based crisis intervention more essential in outpatient care (73.4% “great” or “very great”) compared to inpatient settings (55%).

**Fig. 2:**
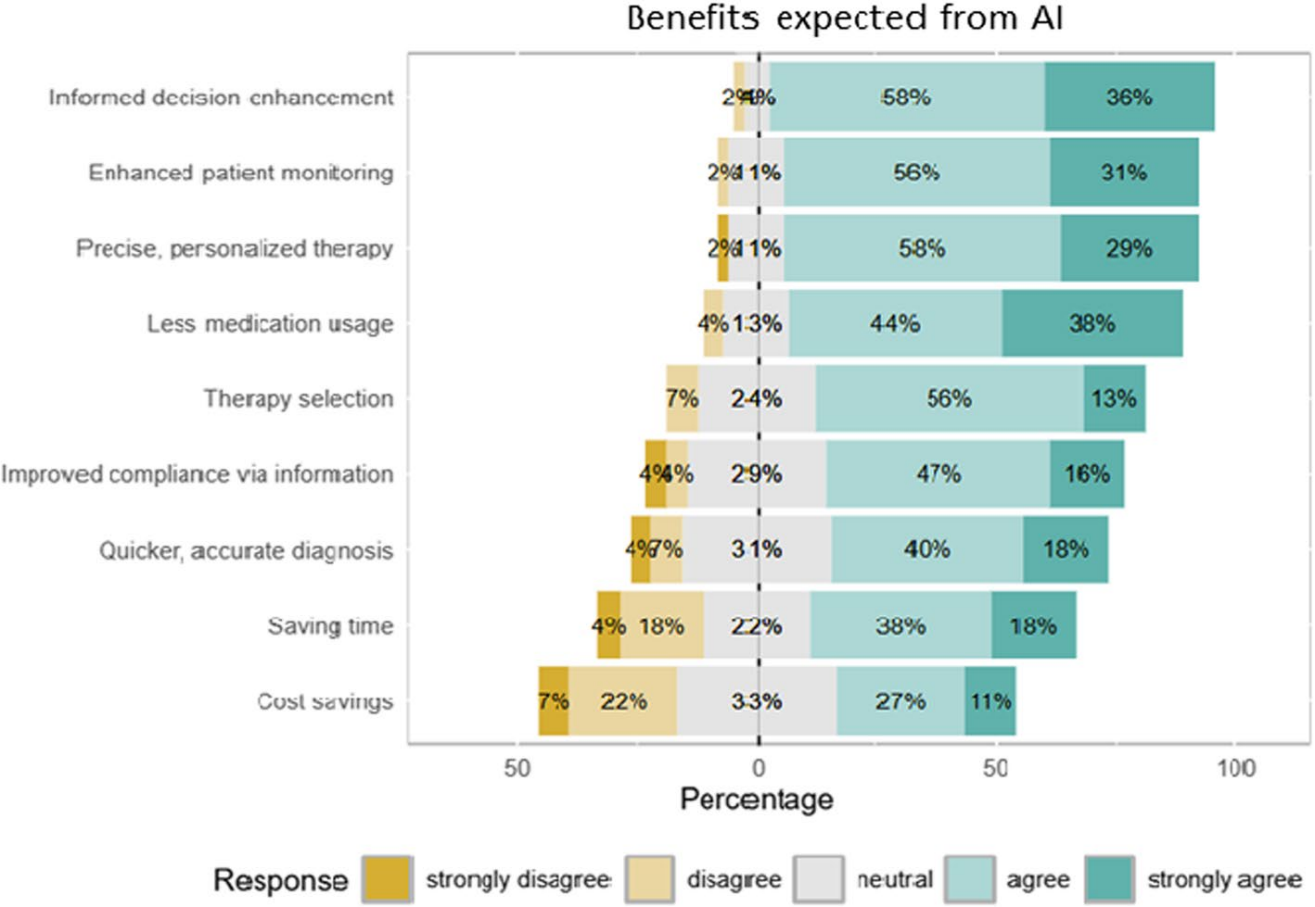
Expected benefits from AI in mental health care as perceived by clinicians. The chart shows the percentage of clinicians who agree or disagree with the potential advantages AI could bring to mental health practice. Highest expectations are placed on *informed decision enhancement*, *enhanced patient monitoring*, and *precise, personalized therapy*. Other valued benefits include *reduced medication usage* and *therapy selection*. Lower priority is given to *time and cost savings*, indicating that clinicians prioritize AI’s role in clinical decision-making and monitoring over operational efficiencies

Clinicians saw better decision-making, enhanced monitoring, and greater precision in treatment choices as primary advantages of AI integration. More accurate, individualized treatment options through AI were also highly valued (Fig. 2).

**Challenges and barriers**. The main barriers to AI adoption identified by clinicians included a lack of confidence in current predictive accuracy (73.3% rated as a concern) and limited time to learn or integrate these tools (64.5%). Technical challenges and inexperience were considered minor issues, as were potential problems with patient dependency or privacy concerns. Interestingly, clinicians with a psychology background expressed greater concern regarding potential losses of empathy and depersonalization resulting from AI use compared to their medical counterparts (mean rank psychology = 26.47, mean rank medicine = 18.14; U = 150; Z = −2.24; $p < 0.025$).

## Discussion

Our findings reveal that clinicians, while aware of AI’s current limitations in mental health [29, 30], are generally optimistic about its potential and appear open to integrating it into practice. Survey responses indicate a readiness among mental health professionals to adopt AI-based tools, particularly for monitoring and prediction, with the two primary barriers identified being concerns about model accuracy and the practical integration of AI into daily workflows [31]. Together, this suggests a strong basis for computational psychiatry to closely integrate EMA methodologies [32], thereby developing tools that directly address real-world clinical needs.

A primary insight from the survey is the emphasis clinicians place on predictive and monitoring capabilities over treatment recommendations or model interpretability. While only 18% rated specific therapy recommendations (e.g., detailed treatment plans) as ‘very important’, clinicians showed greater support for AI-enhancing therapy decisions, with 62% agreeing on ‘precise, personalized therapy’ and 58% on ‘easier therapy selection’. This suggests a preference for AI as a decision-support tool-applicable to both pharmacotherapy and psychotherapy, per survey options like ‘fewer medication trials’ and ‘therapy selection’-rather than a source of prescriptive directives, preserving their role in treatment planning. Clinicians prioritize tools that deliver reliable predictions of patient outcomes and enable real-time monitoring to support proactive decision-making and early identification of at-risk individuals. This preference likely reflects the nuanced nature of psychiatric care, where clinician judgment and patient interaction are essential and will not be replaced by AI [26]. Prediction-oriented tools are particularly appealing, as they provide meaningful insights while respecting the clinician’s domain of treatment decision-making. Clinicians also favor clear, straightforward visualizations-like time-based symptom profiles with intensity markers-over more complex network diagrams or prescriptive treatment suggestions [27]. This aligns with the time constraints many psychiatric practitioners face, ensuring an efficient balance between effort and clinical benefit. Closely related, clinicians are particularly optimistic about AI’s potential to identify high-risk individuals, especially those with suicidal tendencies, where promising predictive methods are already emerging [33, 34]. The survey also highlighted that clinicians with a psychology background are more attuned to patient concerns, emphasizing the importance of a multidisciplinary approach involving clinicians, psychologists, and computational researchers in AI development. Such collaborations are essential to tackle challenges like integrating AI into clinical routine practice, addressing the limited availability of real-world datasets, and managing ethical considerations [13, 35].

The survey also underscores the importance of selecting meaningful data types and optimal collection intervals for effective AI forecasting tools. Established indicators for MDD, such as sleep duration and quality [36–39], physical activity [40, 41], and heart rate variability (HRV) [42, 43], were identified as particularly valuable for prediction. Tracking weight changes [44] was also viewed as feasible and clinically relevant. Among all these variables, however, we find a strong preference for monitoring sleep duration and quality over other biological factors. Interestingly, this aligns very well with major studies on symptom patterns across DSM-5 diagnostic criteria, highlighting sleep problems as the most frequently recurring nonspecific transdiagnostic measure [45]. Notably, clinicians preferred data collection at biweekly intervals, a cadence that aligns with technical recommendations [46] but contrasts with the high-frequency sampling often used in ecological momentary assessment (EMA) studies (e.g., [47]). This insight is valuable for designing practical, clinically aligned AI tools.

In terms of AI accuracy, clinicians indicated that an accuracy threshold of approximately 80% is necessary for AI predictions to be trusted. Achieving this level of reliability may be more feasible when relying on non-neural data sources, as the integration of neural and genetic data into predictive models remains technically challenging and clinically unfamiliar territory [48]. Clinicians identified outpatient settings as the primary context where AI tools could have the most impact, particularly for predicting symptom progression between less frequent follow-up visits. This aligns with the realities of outpatient care, where clinicians have fewer direct patient contacts and would thus significantly benefit from supplementary AI-driven data to monitor risks and symptom trajectories [13, 49]. By contrast, inpatient settings, which offer more regular contact and direct observation, were seen as less dependent on AI-based monitoring. The potential to enhance outpatient care through digital phenotyping—using smartphones and wearable devices to capture real-time behavioral and physiological data—was highlighted as a valuable extension of traditional monitoring. Such technologies could allow clinicians to track subtle shifts in symptom trajectories, enabling earlier intervention [50–52].

Interestingly, the emphasis on 80% accuracy warrants further reflection. This threshold appears to reflect an intuitive benchmark for clinical trust rather than a rigorously defined requirement, particularly given the limited AI training reported by surveyed clinicians. In fact, 44.4% of participants indicated they had no formal training in AI, and only three of the eight interviewed clinicians reported any familiarity with AI applications in mental health practice. This limited exposure suggests that clinicians’ expectations for accuracy might shift with increased education and practical experience. While this 80% may seem modest compared to the higher sensitivity often required of medical devices, it represents an initial trust threshold for AI as a supportive tool, shaped by clinicians’ current familiarity rather than established technical standards. Moreover, while accuracy is an essential criterion, it is not sufficient on its own to evaluate AI models—particularly in psychiatry, where factors such as interpretability, sensitivity to subtle changes, and the clinical relevance of predictions are equally critical [53]. This concern is compounded by the “black box” nature of many AI models, where the lack of transparency in decision-making processes may contribute to clinicians’ reluctance to trust them, as only 27% rated exact prediction accuracy details as ‘very important’, possibly reflecting discomfort with opaque outputs [54]. Approaches like explainable AI (XAI) could address this by offering clearer insights into how predictions are made, potentially increasing acceptance among clinicians wary of current limitations [55]. This underscores the importance of a comprehensive evaluation framework beyond accuracy alone, necessitating a broader dialogue among clinicians, computational psychiatrists, and AI developers. Such a process would require ongoing co-evolution between AI development, computational psychiatry, and real-world clinical practice to ensure these tools meet informed and evolving clinical expectations [13, 26, 56].

This study provides valuable clinician-driven insights into AI priorities for mental health care, but it has limitations that warrant consideration. Notably, many surveyed clinicians reported minimal AI training, which may have shaped their responses, a pattern echoed in prior research [26, 54]. This limited exposure suggests that future surveys should assess clinicians’ AI literacy and define terms like “predictive accuracy” more clearly to refine their expectations. Additionally, the study did not differentiate between neural (e.g., neuroimaging) and non-neural data sources, missing an opportunity to explore perspectives on translationally promising models like those in Nielsen et al. (2020) [57]. Similarly, distinguishing predictive validity from causality could further clarify clinician needs, particularly for actionable tools. The sample size of 53 clinicians, while modest, aligns with exploratory studies in computational psychiatry (e.g. [27]), where samples under 60 have successfully identified key priorities. Our diverse cohort-spanning roles, experience levels, and settings-likely achieved thematic saturation, supporting the study’s aim to map initial clinician expectations. However, this size limits generalizability, and insufficient data prevented robust subgroup analyses (e.g., by experience level). Larger, probability-based samples in future confirmatory research could enhance statistical power and validate these findings, ensuring AI development aligns with evolving clinical demands.

## Conclusions

Our findings reveal a growing openness within the clinical community toward AI for monitoring and predictive tasks, with an emphasis on high prediction accuracy over interpretability or treatment recommendations. This need underscores the importance of computational psychiatry in developing AI tools that combine ecological momentary assessment (EMA) and sleep data to deliver precise, actionable predictions. By prioritizing these clinician-driven preferences, such tools could enhance mental health care by enabling real-time monitoring and early intervention, particularly in outpatient settings where contact is limited, and by improving the identification of high-risk patients, such as those with suicidal tendencies [33, 34]. Meeting these requirements could significantly enhance proactive patient care and bridge critical gaps in mental health services worldwide.

## Supplementary Information

Supplementary Material 1.

## Data Availability

All the data and codes to produce the results will be available publically at https://osf.io/dxqws/.
