# Supplementary material for: AI for mental health: clinician expectations and priorities in computational psychiatry
Source: BMC Psychiatry. 2025 Jun 6;25:584. doi: 10.1186/s12888-025-06957-3 (PMC12143059; doi:10.1186/s12888-025-06957-3)
Supplement: Supplementary file 1 — Supplementary Material 1. [file 12888_2025_6957_MOESM1_ESM.pdf]

Supplementary Material

Supplementary A: The Key Questions Used in the Semi-Structured Interviews

The following section provides the key questions used in the semi-structured interviews conducted with eight clinicians by the authors LF and PM. Each question is presented in both the English translation and the original German version.

1. (English) What is your current professional designation?
   (German) Wie lautet Ihre aktuelle Berufsbezeichnung?

2. (English) How long have you been working in clinical practice?
   (German) Wie lange sind Sie bereits klinisch tätig?

3. (English) Have you had experience working with AI? Have you heard of colleagues who are already utilizing it?
   (German) Haben Sie bereits mit KI gearbeitet? Haben Sie von Kollegen gehört, die damit bereits arbeiten?

4. (English) *Input – what are the potential applications of AI in a psychiatric context?*
   (German) *Input – was kann KI im psychiatrischen Kontext?*

5. (English) What are the specific benefits of the predictions? (How might they impact clinical practice and daily operations?)
   (German) Welchen Nutzen haben die Vorhersagen konkret? (Wie würden sie den Alltag verändern?)

6. (English) Where would the greatest benefit lie: in outpatient or inpatient settings? Which patient groups could potentially benefit the most?
   (German) Wo liegt der größte Nutzen, ambulant oder stationär? Welche Patientengruppen könnten besonders profitieren?

7. (English) Do you regularly administer standardized questionnaires such as the BDI, etc.?
   (German) Erheben Sie regelmäßig Fragebögen wie BDI etc.?

8. (English) Should self-assessments, third-party assessments, or both be utilized? Which is considered most appropriate?
   (German) Sollte man eher Selbst-, Fremdbeurteilungsbögen oder beides verwenden? Welche sind am besten geeignet?

9. (English) Is it feasible to conduct third-party assessments via telephone or video? Can family members fulfill this role?
   (German) Kann man Fremdbeurteilung per Telefon oder Video durchführen? Können Angehörige das leisten?

10. (English) What method would you prefer for data transmission: paper, mobile application, website, or desktop software?
(German) Über welchen Weg würden Sie gerne die Daten weitergeben: Papier, App, Website, PC-Programm?

11. (English) How frequently should scores be collected? (Considering clinically relevant changes and feasibility of implementation)
(German) Wie oft sollte man die Scores erheben? (klinisch relevante Änderung, Umsetzbarkeit)

12. (English) How should the results of the prediction be presented? (Should leading symptoms be included? Should the course of the illness over the past weeks/months/years be considered? Are certain symptoms particularly critical?)
(German) Wie soll die Ergebnispräsentation der Vorhersage aussehen? (führende Symptome dazuschreiben? Verlauf der letzten Wochen/Monate/Jahre dazu? Bestimmte Symptome besonders wichtig?)

13. (English) How could treatment be adapted if the disease trajectory could be predicted?
(German) Wie könnte man die Therapie anpassen, wenn man den Krankheitsverlauf vorhersehen könnte?

14. (English) Would you trust the predictions, and if yes/no, why/why not?
(German) Würden Sie der Vorhersage trauen und wenn ja/nein: Warum/Warum nicht?

15. (English) Should the patient be informed of the prediction results?
(German) Sollte der Patient das Ergebnis der Vorhersage mitgeteilt bekommen?

16. (English) Could this lead to the development of dependency? (For example, would a patient rate themselves more negatively if they know this could result in greater attention?)
(German) Könnte es zu einer Art Abhängigkeit kommen? (z. B.: Patient beurteilt sich selbst schlechter, weil er gelernt hat, dass er dann mehr Aufmerksamkeit bekommt)

17. (English) What factors could hinder the successful implementation of such a system? (e.g., lack of time, unreliable self/third-party assessments, absence of necessary infrastructure, potential exploitation by patients, etc.)
(German) Woran könnte die Umsetzung scheitern? (Zeitmangel, keine zuverlässige Selbst-/Fremdbeurteilung fehlende Strukturen, Ausnutzung durch Patienten...)

18. (English) Would clinical expertise be compromised if excessive reliance is placed on AI?
(German) Geht klinische Expertise verloren, wenn man zu sehr der KI vertraut?

19. (English) Are false-negative predictions potentially harmful to patients (e.g., leading to a negative influence due to the prediction)?
(German) Sind falsch negative Vorhersagen gefährlich für die Patienten (negative Beeinflussung durch Vorhersage?)

20. (English) Could the data be utilized for other purposes, such as therapy monitoring?
(German) Könnte man die Daten noch für andere Zwecke benutzen? (z.B. Therapiekontrolle)

21. (English) Are there other conditions where similar predictive tools could be of benefit?
(German) Gibt es noch andere Krankheiten, bei denen eine ähnliche Vorhersage hilfreich wäre?

Supplementary B: Survey Questions

This supplementary section lists all the questions used in the survey conducted to understand clinicians' expectations and concerns regarding the use of AI in predicting depression trajectories. Each question is presented in both the English translation and the original German version. In the following text, "Sappiens" is used as an acronym for an AI assistant project under development at the Department of Computational Psychiatry, University of Marburg, aimed at predicting symptoms and trajectories in depression. Survey respondents were unaware of this project and encountered "Sappiens" solely as an acronym, accompanied by the explanation "Description of the AI assistant project for predicting symptoms and trajectories in depression," as reflected in the items below.

1. (English) What gender do you identify with?
(German) Mit welchem Geschlecht identifizieren Sie sich?

2. (English) How old are you?
(German) Wie alt sind Sie?

3. (English) What is your current professional designation?
(German) Wie lautet Ihre derzeitige Berufsbezeichnung?

4. (English) How long have you been practicing clinically?
(German) Wie lange sind Sie bereits klinisch tätig?

5. (English) How familiar are you with artificial intelligence in a clinical psychiatric context?
(German) Wie gut kennen Sie sich bereits mit künstlicher Intelligenz im klinischen, psychiatrischen Kontext aus?

6. (English) What is the purpose of Sappiens? (Explanation: Description of the AI assistant project for predicting symptoms and trajectories in

depression)
(German) Worum geht es bei Sappiens? (Erklärung: Beschreibung des KI-Assistentenprojekts zur Vorhersage von Symptomen und Krankheitsverläufen bei Depressionen)

7. (English) In your opinion, what would be an appropriate time interval for administering the following questionnaires to depression patients: BDI, PHQ9, Hamilton?
   (German) Zur Symptomerhebung mussten die Depressions-Patient*innen regelmäßig folgende Fragebögen ausfüllen: BDI, PHQ9, Hamilton. Was ist Ihrer Meinung nach dafür ein geeignetes Zeitintervall?

8. (English) How reliable do you think the following groups are in making third-party assessments of patients? (Attending clinicians, attending nursing staff, close relatives, close relatives selected individually by clinicians, legal guardians/caretakers)
   (German) Wie zuverlässig können Ihrer Meinung nach die folgenden Gruppen eine Fremdbeurteilung von Patient*innen vornehmen? (Betreuende Kliniker*innen, betreuendes Pflegepersonal, enge Angehörige, enge Angehörige nachdem Kliniker*innen diese individuell ausgewählt haben, Vormund/Betreuer)

9. (English) How suitable do you consider the following methods of patient contact for attending clinicians to reliably complete a third-party assessment? (In-person contact with the patient, video conference, telephone call)
   (German) Wie gut sind Ihrer Meinung nach die folgenden Methoden von Patient*innenkontakt für betreuende Kliniker*innen geeignet, um einen Fremdbeurteilungsbogen zuverlässig auszufüllen? (Kontakt in Anwesenheit der/des zu beurteilenden Patient*in, Videokonferenz, Telefonat)

10. (English) How appropriate do you believe the following physical parameters are for improving the accuracy of predicting the disease course in depressive patients? (Sleep quality, sleep duration, physical activity, number and amount of meals per day)
    (German) Wie geeignet ist Ihrer Einschätzung nach die Erfassung der folgenden körperlichen Parameter zur Verbesserung der Genauigkeit der Vorhersage des Krankheitsverlaufs bei depressiven Patient*innen? (Schlafqualität, Schlafdauer, Bewegungsumfang, Anzahl und Menge der Mahlzeiten pro Tag)

11. (English) How important are the following factors to you when presenting the results of the prediction regarding the disease trajectory? (Central symptoms, intensity of current symptoms, duration of current symptoms, previous course of illness, current suicidality, perceived accuracy of the prediction, therapy recommendation, network graph illustrating symptom interrelations)

(German) Wie wichtig sind Ihnen als Behandler*in die nachfolgenden Informationen bei der Ergebnispräsentation zur Vorhersage des Krankheitsverlaufs? (Zentrale Symptome, Intensität der aktuellen Symptomatik, Dauer der aktuellen Symptomatik, bisheriger Krankheitsverlauf in Kurvenform, aktuelle Suizidalität, vermutete Präzision der Vorhersage, Therapieempfehlung, Netzwerkgrafik zur gegenseitigen Beeinflussung der Symptome)

12. (English) Imagine you are using our AI assistant in clinical practice and receive a prediction for one of your outpatient patients indicating that their disease course is likely to worsen in the near future. How would you proceed next? (Phone call with the patient, in-person meeting with the patient, adjustment of medication, adjustment of psychotherapy, no adjustment)
(German) Stellen Sie sich vor: Sie benutzen unseren KI-Assistenten im klinischen Alltag und bekommen jetzt für eine/n Ihrer ambulanten Patient*innen die Vorhersage, dass sich der Krankheitsverlauf in nächster Zeit vermutlich verschlechtern wird. Wie würden Sie als nächstes vorgehen? (Telefongespräch mit Patient*in, persönlicher Kontakt mit Patient*in, Anpassung der Medikation, Anpassung der Psychotherapie, keine Anpassung)

13. (English) Would you inform your patients of the predicted disease course result? (Provided that the patients agree, as part of "informed consent," to be informed of the results)
(German) Würden Sie Ihren Patient*innen das Ergebnis der Vorhersage des Krankheitsverlaufs mitteilen? (Vorausgesetzt, die Patient*innen sind im Rahmen des "informed common sense" einverstanden, dass Ihnen die Ergebnisse mitgeteilt werden)

14. (English) How do you evaluate the following reasons for informing patients of the prediction result? (To strengthen trust between patient and clinician, demonstrate transparency, improve compliance, enhance psychoeducation, encourage proactive changes, improve long-term course of disease)
(German) Wie beurteilen Sie die folgenden Gründe, den Patient*innen das Ergebnis der Vorhersage mitzuteilen? (Vertrauen zwischen Patient*in und Behandler*in stärken, Transparenz zeigen, Compliance erhöhen, Psychoedukation stärken, proaktive Veränderungen bewirken, Krankheitsverlauf langfristig verbessern)

15. (English) How do you evaluate the following reasons for not informing patients of the prediction result? (Patients might refrain from health-promoting behaviors, false-negative predictions could worsen disease course, time investment for clinicians is too great, emotional burden on patients)
(German) Wie beurteilen Sie die folgenden Gründe, den Patient*innen das Ergebnis der Vorhersage nicht mitzuteilen? (Patient*innen könnten gesundheitsfördernde Maßnahmen unterlassen, falsch negative Vorhersagen

könnten den Krankheitsverlauf verschlechtern, Zeitaufwand für Behandler*innen zu groß, emotionale Belastung der Patient*innen zu stark)

16. (English) How important are the following factors when deciding whether or not to share the prediction result with the patient? (Emotional stability, patient's wishes, severity of the condition, type of result, compliance, assessment of self-reflection, type of contact, type of illness)
(German) Wie wichtig sind Ihnen die folgenden Kriterien in der Entscheidung, ob den Patient*innen das Ergebnis der Vorhersage mitgeteilt werden sollte? (Emotionale Stabilität, Patient*innenwunsch, Schweregrad der Erkrankung, Art des Ergebnisses, Compliance, Einschätzung der Selbstreflexion, Art des Kontaktes, Art der Erkrankung)

17. (English) How problematic could the following factors be in the implementation of our project? (Poor cost-benefit ratio, lack of trust in predictions, time constraints, unreliable self-assessment data, insufficient data protection, technical issues, risk of dependency in patients)
(German) Wie problematisch könnten Ihrer Meinung nach die folgenden Faktoren in der Umsetzung unseres Projektes sein? (Aufwand-Nutzen-Verhältnis zu gering, zu wenig Vertrauen in die Vorhersage, Zeitmangel, unzuverlässige Selbstbeurteilung, Datenschutz nicht gewährleistet, technische Probleme, Risiko der Abhängigkeit bei Patient*innen)

18. (English) How do you evaluate the following statements regarding the use of AI in the context of Sappiens? (Clinical expertise is lost, patient privacy is compromised, AI leads to depersonalization, loss of human care, errors are a concern, time savings for clinicians, improved decision-making, better monitoring, more precise therapy, faster diagnosis, improved compliance, easier therapy selection, cost savings)
(German) Wie bewerten Sie die folgenden Aussagen zum Einsatz von künstlicher Intelligenz im Rahmen von Sappiens? (Klinische Expertise geht verloren, Datenschutz gefährdet, KI führt zu Depersonalisierung, Verlust menschlicher Zuwendung, Fehler sind ein großes Bedenken, Zeitersparnis für Kliniker*innen, verbesserte Entscheidungsfindung, besseres Monitoring, präzisere Therapie, schnellere Diagnose, verbesserte Compliance, leichtere Therapieauswahl, Kosteneinsparungen)

19. (English) How beneficial would a prediction of the disease course by AI be in the following areas? (Outpatient: crisis prevention, therapy monitoring; Inpatient: course monitoring, therapy monitoring)
(German) Wie groß wäre der Nutzen einer Vorhersage des Krankheitsverlaufs durch KI in den folgenden Bereichen? (Ambulant: Krisenprophylaxe, Therapiekontrolle; Stationär: Verlaufsbeobachtung, Therapiekontrolle)

20. (English) How beneficial would the prediction tool be for the following professional groups? (General practitioners, private practice psychotherapists, psychiatrists, neurologists, psychology students, psychotherapists in training, medical students, resident physicians, specialist physicians,

inpatient psychologists)
(German) Wie groß wäre der Nutzen des Vorhersage-Tools für die folgenden Berufsgruppen? (Hausärzt*innen, niedergelassene Psychotherapeut*innen, Psychiater*innen, Neurolog*innen, Psychologiestudent*innen, Psychotherapeut*innen in Ausbildung, Medizinstudent*innen, Assistenzärzt*innen, Fachärzt*innen, stationär tätige Psycholog*innen)

21. (English) At what level of prediction accuracy would you trust the AI assistant and consider its use advantageous?
(German) Ab welcher Präzision würden Sie dem KI-Assistenten vertrauen und seinen Einsatz als vorteilhaft bewerten?

22. (English) What benefits do you hope to gain from the use of AI in the clinical psychiatric context? (Time savings, fewer medication trials, better decision-making, improved monitoring, more precision, faster diagnosis, improved compliance, easier therapy selection, cost savings)
(German) Welche Vorteile erhoffen Sie sich durch den Einsatz von KI im klinischen psychiatrischen Kontext? (Zeitersparnis, weniger medikamentöse Therapie-Versuche, bessere Entscheidungsfindung, besseres Monitoring, präzisere Therapie, schnellere Diagnose, bessere Compliance, leichtere Therapieauswahl, Kosteneinsparungen)

23. (English) Are there other conditions or patient groups where predicting the disease course would be useful?
(German) Fallen Ihnen (neben Depressionen) noch weitere Krankheiten oder Patient*innengruppen ein, bei denen eine Vorhersage des Krankheitsverlaufs nützlich wäre?

24. (English) What would your ideal AI assistant look like? What options and content would it include? How would it assist you in your clinical practice?
(German) Wie sähe Ihr perfekter KI-Assistent aus? Welche Auswahlmöglichkeiten und Inhalte hätte er? Wobei würde er Ihnen im klinischen Alltag helfen?
